# Molecular and Genomic Insights of *mcr-1*-Producing *Escherichia coli* Isolates from Piglets

**DOI:** 10.3390/antibiotics11020157

**Published:** 2022-01-26

**Authors:** Jonathan Rodríguez-Santiago, Nadia Rodríguez-Medina, Elsa María Tamayo-Legorreta, Jesús Silva-Sánchez, Juan Téllez-Sosa, Josefina Duran-Bedolla, Alejandro Aguilar-Vera, Alba Neri Lecona-Valera, Ulises Garza-Ramos, Celia Alpuche-Aranda

**Affiliations:** 1 Centro de Investigación Sobre Enfermedades Infecciosas (CISEI), Instituto Nacional de Salud Pública (INSP), Cuernavaca 62100, Morelos, Mexico; rguez.jonathan@gmail.com (J.R.-S.); nadia_yeli@hotmail.com (N.R.-M.); emtamayo@insp.mx (E.M.T.-L.); jsilva@insp.mx (J.S.-S.); jmtellez@insp.mx (J.T.-S.); josefina.duran@insp.mx (J.D.-B.); alba.lecona@insp.mx (A.N.L.-V.); 2 Programa de Genómica Funcional de Procariotes, Centro de Ciencias Genómicas, Universidad Nacional Autónoma de México, Cuernavaca 62100, Morelos, Mexico; aaguilar@ccg.unam.mx

**Keywords:** ESBL-producing, colistin, colistin resistance, plasmid, *mcr-1*, phage, piglet

## Abstract

The use of colistin in food-producing animals favors the emergence and spread of colistin-resistant strains. Here, we investigated the occurrence and molecular mechanisms of colistin resistance among *E. coli* isolates from a Mexican piglet farm. A collection of 175 cephalosporin-resistant colonies from swine fecal samples were recovered. The colistin resistance phenotype was identified by rapid polymyxin test and the *mcr*-type genes were screened by PCR. We assessed the colistin-resistant strains by antimicrobial susceptibility test, pulse-field gel electrophoresis, plasmid profile, and mating experiments. Whole-Genome Sequencing data was used to explore the resistome, virulome, and mobilome of colistin-resistant strains. A total of four colistin-resistant *E. coli* were identified from the cefotaxime-resistant colonies. All harbored the plasmid-borne *mcr-1* gene, which was located on conjugative 170-kb IncHI-2 plasmid co-carrying ESBLs genes. Thus, high antimicrobial resistance rates were observed for several antibiotic families. In the RC2-007 strain, the *mcr-1* gene was located as part of a prophage carried on non-conjugative 100-kb-plasmid, which upon being transformed into *K. variicola* strain increased the polymyxin resistance 2-fold. The genomic analysis showed a broad resistome and virulome. Our findings suggest that colistin resistance followed independent acquisition pathways as clonal and non-genetically related *mcr-1*-harboring strains were identified. These *E. coli* isolates represent a reservoir of antibiotic resistance and virulence genes in animals for human consumption which could be potentially propagated into other interfaces.

## 1. Introduction

Colistin represents the last line of therapeutic options against multidrug-resistant strains of Enterobacterales. However, unregulated overuse of colistin in the animal sector as a growth promoter and for preventing infections in food-producing animals has favored the emergence of colistin-resistant strains [1]. Colistin resistance is associated with LPS lipid A modifications, which can be attributed to either chromosomal mutations in regulatory two-component systems or the plasmid-borne *mcr* gene variants [2].

Plasmid-borne *mcr* genes have been found in a variety of plasmid types such as IncI2, IncHI2, IncX4, IncP, IncF and the less common IncY [3]. These plasmids are transferable with a broad host range, which could explain the widespread of *mcr* genes among different bacterial species [4]. Ten variants of the *mcr* gene have so far been described [5], but the most widespread is the *mcr-1* gene [6]. This gene has been identified in isolates from numerous sources including humans, food, farms and wild animals [7,8]. It has been demonstrated that the transfer of the *mcr-1* gene in enterobacteria from farm animals to humans through the food chain [9]. Notably, *mcr* genes are found in association with other antimicrobial resistance genes, usually extended-spectrum-ß-lactamases (ESBL) and carbapenemases, highlighting the emergence of pan-drug resistant Enterobacterales [6].

Moreover, phages also play a role in the dissemination of antimicrobial resistance genes [10]. Some phages have the ability to integrate into plasmids and can be transferred to other bacteria [11]. Particularly, phage-like plasmids carrying the *mcr-1* gene have been described in *Klebsiella pneumoniae* and *Escherichia coli* [3,12].

In Mexico, polymyxins, as well as other antibiotics, have not been approved as growth promoters since 2012, and there are few reports of isolates carrying the *mcr-1* gene [6]; nevertheless, this gene has been identified from clinical [13] and swine farm [14] environments. In addition, it has also been described in cantaloupes [15]. This study provides insight into the molecular mechanisms of colistin resistance circulating in a Mexican piglet farm and by genomic analysis, we explore the resistome, virulome and mobilome of *mcr-1*-producing *E. coli* isolates.

## 2. Results

### 2.1. Occurrence of Colistin Resistance on a Swine Farm

Of the stool samples plated on agar plates supplemented with cefotaxime, we randomly selected one colony for each plate. The frequency of cefotaxime-resistant colonies was 34.4% (175/508). Among 175 cefotaxime-resistant colonies, four colistin-resistant (C072, C2-033, C2-107 and C2-108) were identified by rapid polymyxin NP test and were confirmed as *E. coli* according to the biochemical test kit API20E. The plasmid-borne *mcr-1* gene was identified in the four *E. coli* colistin-resistant isolates (Table 1). The C072 isolate was collected in March 2015 and the C2-033, C2-107 and C2-10 isolates, in September 2015. All *mcr-1*-positive isolates including the RC2-007 isolate showed a colistin MIC of 4 μg/mL. The RC2-007 isolate was previously described from the same farm among 928 *E. coli* strains [14]. PFGE analysis demonstrated that C2-107 and C2-108 isolates were the same clone and the rest were genetically unrelated. All colistin-resistant isolates were also identified as ESBL-producers and were resistant to ampicillin, ceftazidime, cefotaxime, gentamicin, nalidixic acid and tetracycline but susceptible to imipenem, amikacin and ciprofloxacin. The RC2-007 isolate, however, showed greater resistance to ceftazidime, gentamicin and ciprofloxacin (Table 1).

### 2.2. Plasmid Analysis of mcr-1-Harboring E. coli Isolates

The plasmid-borne *mcr-1* gene was identified on a ∼170-kb plasmid shared among the C072, C2-033, C2-107 and C2-108 isolates (Table 1). The mating experiments were successful in the isolates that carried the 170-kb plasmid with different conjugation efficiencies (Table 1). In contrast, as the RC2-007 was unable to mobilize the *mcr-1* gene, we performed transformation experiments using the *E. coli* DH10B and *K. variicola* F2R9 isolates as receptors (to evaluate the *mcr-1* gene in a different genetic background). The plasmid that was successfully transferred in both receptors (TpEcoDH10B and TpKvF2R9) was the ~100-kb plasmid (Table 1).

The transconjugants T-C072, T-C2033, T-C2-107, T-C2-108 were confirmed to have decreased colistin susceptibility by MIC determination. Transconjugants exhibited a MIC for colistin of 2 and 1 μg/mL; otherwise, the TpEcoDH10B and TpKvF2R9 transformants exhibited a MIC 2 and 16 μg/mL, respectively (Table 1). In addition, all *mcr-1* positive isolates also were ESBL-producers and in all cases the ESBLs CTX-M-type were identified by PCR amplification (Table 1). In the C072, C2-033, C2-107 and C2-108 isolates the CTX-M-type genes are contained in the same plasmid as the *mcr-1* gene. In contrast, the colistin and cephalosporin resistance exhibited by the RC2-007 isolate were encoded on different plasmids (Table 1).

### 2.3. Whole Genome Analysis of mcr-1-Producing E. coli Isolates

Genome features of C072, C2-033, C2-107 and C2-108 isolates are described in Supplementary Table S1. The C072 and C2-033 isolates belonged to distinct sequence types (ST), and the isolates C2-107 and C2-108 were ST1286. In the same way, serotypes varied across the strains. ClermonTyper determined that the majority of the *mcr-1*-positive strains were grouped into phylogroups from commensal strains (group A or B1) but C2-033 belonged to group F (Table 2).

**Figure 1:**
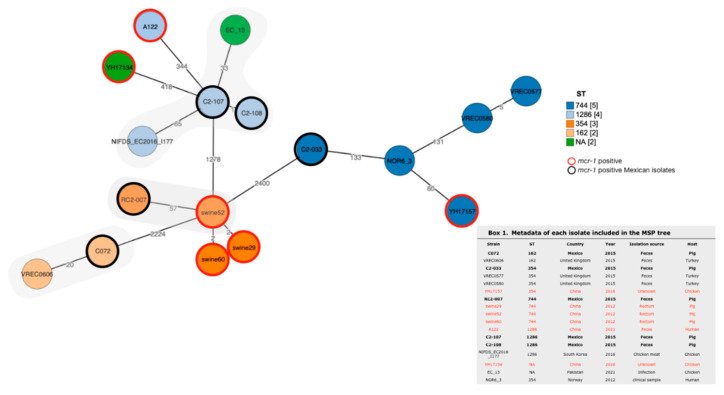
Minimum spanning tree based on cgMLST comprising 2513 genes of *E. coli*. Each group is formed by isolates with the same ST as marked by color code. This analysis includes both *mcr-1*-producing and -non-producing but genetically related to C072, C2-107, C2-108, C2-033 and RC2-007. Numbers between lines indicate allele differences between isolates. Gray shadows represent closely related isolates. An information box containing metadata for each isolate is included.

In order to explore the genetic relationship of the five *mcr-1* positive isolates from this study, we compared them with *E. coli* isolates from other sources and/or isolates carrying the *mcr-1* gene that have the same ST. For this purpose, we performed a minimum spanning tree (MSP) based on cgMLST (Figure 1). Using a threshold of less than 25 allele differences, only the C072 isolate was found to be genetically related to the VREC0606 isolate derived from turkey feces: this isolate was not *mcr-1*-producing *E. coli.* Then, by using a threshold of 50 and 100 allele differences, we identified that C2-107 and C2-108 demonstrated a 33-allele difference to the closest EC_13 isolate, derived from chicken infection and the RC2-007 isolate was related with isolates from pig rectum which were *mcr-1*-producing. No close genetic relationship was found for the C2-033 isolate.

**Figure 2:**
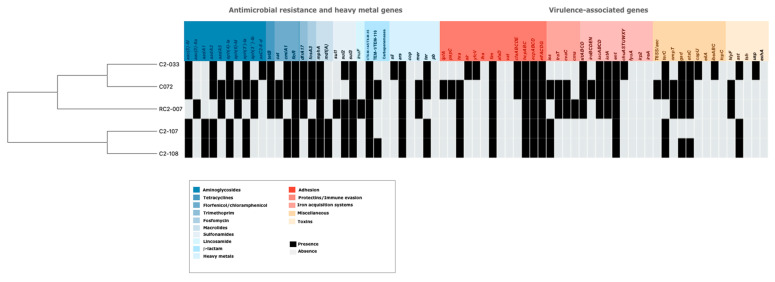
Repertoire of antimicrobial resistance and virulence genes among *mcr-1*-producing *E. coli* isolates from fecal piglet samples. Blue colors represent different antibiotic families and red colors are indicative of virulence-associated genes involved in pathogenesis. Abbreviations for heavy metals correspond to sil: silver, ars: arsenate, cop: copper, mer: mercury, ter: tellurium, and pb: lead.

Consistent with their multi-drug resistant phenotype, the isolates showed an extensive resistome, with genes and chromosomal mutations that confer resistance to several antibiotic families as aminoglycosides, tetracyclines, florfenicol, chloramphenicol, trimethoprim, fosfomycin, macrolides, sulfonamides, lincosamide, β-lactam, quinolones and fluoroquinolones (Figure 2). The main ESBLs corresponded to CTX-M-14, followed by CTX-M-55, and TEM-116. In all isolates the chromosomal mutations in the quinolone resistance-determining region (QRDR) in GyrA and ParC proteins were identified (Supplementary Table S2). Genes encoding for resistance to some heavy metals were found except for copper and lead (Figure 2). Replicon typing showed diverse incompatibility groups (Table 2), the IncHI2 and IncN were shared among C072, C2-033, C2-107 and C2-108 isolates; however, the Incp0111 was identified in the RC2-007 isolate (Table 2).

**Figure 3:**
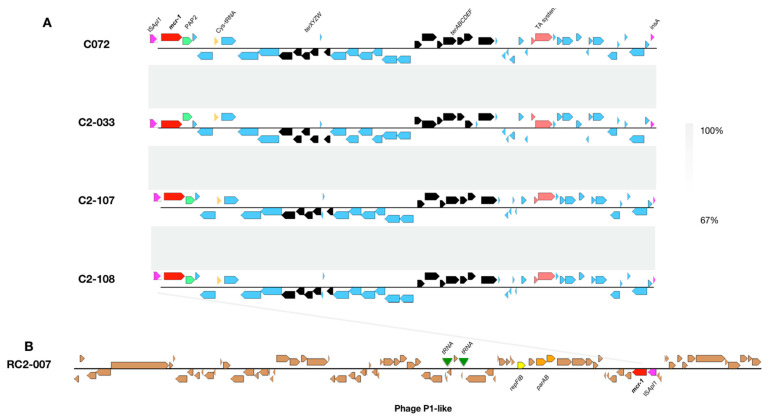
(**A**). Genetic contexts of the *mcr-1* gene among *E. coli* from piglet fecal samples. Light grey shadows show BLAST identity percentage. (**B**). Genetic structure of the phage P1-like carrying the *mcr-1* gene. Each CDS is represented as an arrow frame.

The genetic context of the *mcr-1* gene was analyzed; however, the IS*ApI1* upstream of *mcr-1*-PAP2 genes could not be resolved by whole genome sequencing (except for RC2-007), thus we verified its presence by PCR and sequencing (Table 2). The whole-genome analysis revealed that the IS*ApI1*-*mcr-1*-PAP2 genetic structure in the 170-kb plasmid from C072, C2033, C2-107 and C2-108 is conserved across these isolates (Figure 3). For these isolates, downstream from the IS*ApI1*-*mcr-1*-PAP2 region we found a Cys-tRNA, the tellurium resistance cluster, and the HigAB toxin-antitoxin system (Figure 3). No other antimicrobial resistance genes were found in this region. However, we detected genes conferring resistance to aminoglycosides, chloramphenicol, florfenicol and quaternary ammonium located in class I integron (data not shown). In the case of the RC2-007 isolate the *mcr-1* gene was contained in an intact phage of 79,052 bp identified in a contig of 80,111 bp. In this contig, we also found the *pdh/doc* toxin-antitoxin system (data not shown).

Finally, 40 virulence-associated genes that are present in the ExPEC pathotype were screened in the five *mcr-1*-harboring strains. The highest numbers of ExPEC virulence factors were detected in C2-033 and C072 (Figure 2). However, C072 possessed more virulence genes (22/40) than the remaining strains whereas C2-107 and C2-108 contained less virulence factors (Figure 2). Mobile genetic elements (MGEs) linked with some virulence genes were identified in the C072 and RC2-007 isolates. In the case of C072, we found that *ompT*, *etsC*, and *hlyF* virulence genes were located within the IncFIB plasmid and were associated with the *IS*629; in contrast, *papC*, *terC* and *hra* were identified in the chromosome possibly located in a genomic island and associated with *IS*630 and *IS*609, respectively. Similarly, the RC2-007 contained the virulence genes *hlyF*, *ompT*, *cvaC*, and *cma,* within an IncFIB plasmid in which the CTX-M-55 was also detected. Other MGEs were detected like the *IS*629 linked with the Hra adhesin and the *IS*Ec9 linked with protectins.

### 2.4. In Vitro and In Silico Analysis of Phage Carrying mcr-1

Mitomycin C treatment of the *E. coli* RC2-007 isolate resulted in phage induction. The phage was purified and analyzed by electronic microscopy (Supplementary Figure S2). However, purified genetic material was negative for the *mcr-1* gene by PCR, and consequently the induced phage did not correspond to the phage-like plasmid carrying the *mcr-1* gene. The induced phage could correspond to a phage integrated into the chromosome or another plasmid.

The genetic structure of the phage-like plasmid on the pRC2-007 (100-kb) plasmid was compared with other phage-like plasmids carrying *mcr-1* genes (Supplementary Figure S3). Two *E. coli* isolates (WZR78, GD27-62) [16] and one *K. pneumoniae* (F160070) isolate (MG288678.1) were selected based on BLAST identity. The isolates *E. coli* WZR78 harboring the pZR78 plasmid and GD27-62 harboring the pGD27-62 plasmid were recovered from a pig farm and chicken gut respectively. The *K. pneumoniae* F160070 harboring the p160070-MCR plasmid was recovered from food in China. The genetic comparison among these phage-like plasmids carrying the *mcr-1* gene showed a >95% nucleotide identity and the pZR78 (91,281 bp), GD27-62 (92,362 bp) and p160070-MCR (97,393 bp) with similar molecular sizes also have a >95% identity. We did not identify additional resistance genes in the pRC2-007 (100-kb) plasmid. However, the *E. coli* RC2-007 was described as an MDR isolate and an additional conjugative 120-Kb plasmid that transfers the cephalosporin resistance was described. Otherwise, a P7 phage-like plasmid carrying the *mcr-1* gene was described in a *K. pneumoniae* clinical isolate. The *mcr-1* was carried by a 97.4-kb pMCR_SCKPLL83 plasmid, which did not carry any additional known antimicrobial resistance genes. The conjugation and transformation of pMCR_SCKPLL83 plasmid were unsuccessful using *E. coli* as recipient cells.

### 2.5. Phages and Prophages Carrying mcr-1 Gene in Public Databases

We sought to determine the distribution of phages and prophages harboring the *mcr-1* gene from genome assemblies deposited in the GenBank database. A total of 13,557 and 6603 *E. coli* and *K. pneumoniae* genomes were obtained from the RefSeq database, respectively (Supplementary Table S3). In *E. coli*, 689 and 12,868 with the level of complete and draft genomes, respectively, were identified. Meanwhile, in *K. pneumoniae* 306 complete and 6297 draft genomes were identified. The *mcr-1* gene was detected in a total of 868 (6.4%) of *E. coli* and 45 of *K. pneumoniae* (0.6%) genomes. The *in-silico* prediction of phages and prophages carrying the *mcr-1* gene in *E. coli* genomes were 10 intact (Cat-1) and 12 incomplete (Cat-2) phages. Nevertheless, both phages (342) and prophages (214) in the categories Cat-3 and Cat-6 (questionable) were identified. In addition, four incomplete prophages (Cat-5) were also identified in *E. coli* (Supplementary Table S3). A total of 30 phages and prophages carrying the *mcr-1* gene in their different categories were identified in *K. pneumoniae* (Supplementary Table S3). An intact (Cat-1) and an incomplete (Cat-2) phage were identified, corresponding to SCKP-LL83 and SCKP020138 isolates. Likewise, as in *E. coli*, the number of questionable phages (25) and prophages (3) were mostly identified in *K. pneumoniae*. These data showed that at least in *E. coli* genomes, a high percentage contain the *mcr-1* gene. Likewise, the identification of phages or prophages in any of the categories that contain the *mcr-1* gene is high (59.6%—518/868). This percentage of positive-*mcr-1* genomes decreases in *K. pneumoniae*, although the percentage of phages and prophages carrying the *mcr-1* gene is high (66%—30/45). The replicon containing the phages and prophages carrying the *mcr-1* gene in their different categories was not determined.

## 3. Discussion

In this work, we performed a survey of fecal isolates from a swine farm that is exclusively dedicated to the sale and breeding of piglets. As *mcr-1* co-occurs frequently with cephalosporin-resistance phenotype [17], we cultured the fecal samples on a medium supplemented with cefotaxime. Among the cefotaxime-resistant isolates, four *E. coli* colistin-resistant were identified as being carriers of the plasmid-borne *mcr-1* gene. In addition, we included the colistin-resistant and ESBL-producing RC2-007 isolate previously described in the same farm [14]. Our results suggest that the prevalence of *E. coli* isolates carrying the *mcr-1* gene is low, and is higher in ESBL-producing isolates. The *mcr-1*-positive isolates were obtained only from piglets (Table 1), whereas the adult females and stallions were negative for colistin-resistant *E. coli* strains. Further, we collected one *mcr-1*-carrying isolate in march 2015 (C-072) and the other four in September 2015; which suggests that despite the low prevalence, the dissemination of the *mcr-1* is maintained in the farm, especially in pigs with a few days old.

Colistin has been widely used in poultry and pig farms and its use is linked with high rates of resistance [6]. However, in this work the occurrence of colistin resistance was low; this may imply that colistin is not used on the animals as an antimicrobial treatment or growth promoter. As we could not find *mcr-1*-colonizing strains from the mother, this may suggest that the acquisition of colistin-resistant strains comes from other sources in the environment. For the previously described RC2-007 isolate, the genetic context of the *mcr-1* gene was found in a phage contained on a non-conjugative plasmid; however, by mitomycin C treatment we could not induce this phage. This plasmid could transfer the colistin resistance phenotype by transformation; moreover, transformants exhibited a high MIC for *K. variicola* (Table 1). This data suggest that the *mcr-1* gene could confer greater resistance to colistin in this bacterial species. Colistin-resistant *K. variicola* isolates have been described [18], and recently one isolate of *K. variicola* positive for the *mcr-1* gene was identified in China [19].

Here, three *mcr-1* positive isolates were genetically unrelated and two were clonally related as demonstrated by PFGE analysis and the MSP tree (Figure 1 and Table 1); thus, colistin resistance followed independent acquisition pathways: plasmid-mediated, clonal dissemination, and phage-mediated (Figure 1 and Table 1). However, the self-transmissible 170-kb IncHI2 plasmid had a major distribution in the *mcr-1* positive isolates (Table 1). This is consistent with the fact that the spread of the *mcr-1* gene in Latin America seems to be more related to the spread of *mcr-1* carrying plasmids among *E. coli* isolates than to the clonal expansion of MCR-1 producing isolates [6]. The most prevalent *mcr-1* carrying plasmids in Latin America are the IncX4- and IncI2-types. These Inc-types were not identified in this study. Nevertheless, the IncI2 was reported in a clinical isolate from Mexico [13]. The spreading of the *mcr-1* gene in *E. coli* from the veterinary sector in Mexico could be determined by the dissemination of plasmids different from those in the clinical setting, such as the IncHI2-type plasmids [6]. Interestingly, we identified toxin-antitoxin systems in the genetic contexts of all *mcr-1* positive isolates, suggesting that these plasmids could be maintained even in the absence of selection pressure.

To place in a global context, the five *mcr-1*-positive isolates were compared with *E. coli* isolates from other regions. The results support the role of the *mcr-1*-carrying plasmid, as we identified a genetic relationship with non *mcr-1*-producing isolates from turkey and chicken (Figure 1). Only one *E. coli* isolate (RC2-007) was closely related to an *mcr-1*-producing *E. coli* isolate (swine52), but its mechanism may not be associated with a phage-like plasmid (data not shown)

Unfortunately, we were not provided information about antibiotics administered, age of piglet sale, strategies of waste management or cleaning, but piglets are fed with food enriched with enrofloxacin probably to prevent urinary or intestinal infections. This seems to be related to resistance to quinolone and fluoroquinolones (Table 1 and Figure 2).

The presence of virulence-associated genes such as those coding for colonizing factors (fimbriae and adhesins), survival of bacterial cells (protectins and siderophores) or causing the host inflammatory response, e.g., toxin production [20] were determined in the five *mcr-1*-harboring *E. coli* (Figure 2). The isolates C072 and C2-033 exhibited more virulence-associated genes; this is consistent with their phylogroups, B1 and F, respectively. The strains from phylogroup F are considered highly virulent pathotypes and phylogroup B1 are intestinal pathogenic *E. coli* [21,22]. Moreover, the C072 isolate showed genes coding for a TSS6, which has an important role on virulence (Figure 2) [23].

The RC2-007 also possessed a vast distribution of ExPEC virulence factors (Figure 2). Although phylogroup A is associated with commensal strains, some pathogenic *E. coli* fall into group A, and this might be the case for RC2-007. Moreover, some virulence factors were found on IncFIB plasmids in some cases associated with insertion sequences; this shows that IncFIB plasmids are disseminating antibiotic resistance such as CTX-M-type and virulence genes. Correlation between virulence and antimicrobial resistance has been observed in *mcr-1* positive strains [24]. Finally, our results showed that phages carrying the *mcr-1* gene are more common in *E. coli* than in *K. pneumoniae.* A deeper analysis of the genomic structure of the phages as important vehicles for disseminating colistin resistance deserves study.

Although we found a few colistin-resistant strains probably from environmental contamination by *mcr-1*-harboring *E. coli*, this represents a reservoir of antibiotic resistance and virulence genes in animals for human consumption that could be potentially propagated into other interfaces. Finally, the *mcr-1-*producing *E. coli* strains fall into virulent pathotypes which could represent harm to human health.

## 4. Material and Methods

### 4.1. Sampling and Bacterial Selection

A semi-tech swine farm located in Jiutepec, Morelos at 90 km from Mexico City was selected to carry out this study. The sampling was performed in the total swine population at two stages, stage 1 in March 2015 and stage 2 in September 2015. Overall, 280 swine from stage 1 (163 piglets, 112 females and five stallions) and 228 from stage 2 (123 piglets, 99 females and six stallions) were sampled. In each stage the swine stools were collected and transported in Cary-Blair medium (DELTALAB, Barcelona, Spain) at 4 °C. All stool samples (508) were cultured on MacConkey agar plates supplemented with 1 µg/mL of cefotaxime at 37 °C for 24 h.

The RC2-007 isolate was recovered from the same farm but it was part of a different bacterial collection [14]. At that time (2016), phenotypic tests for colistin resistance detection were not available, and so screening of potential colistin-resistant strains in that particular collection was performed directly by PCR [14]. The RC2-007 isolate was further characterized together with the colistin-resistant isolates found in the present study.

### 4.2. Identification of Colistin Resistant and Screening of Colistin and Cephalosporin Resistant Genes

All *E. coli* phenotype colonies were analyzed using the Rapid Polymyxin NP assay [25] by determining the colistin-resistant phenotype. The plasmid-borne *mcr-1*, *mcr-2*, *mcr-3*, *mcr-4* and *mcr-5* genes and the ß-lactamases of the family CTX-M-type genes were screened by PCR using generic primers (Supplementary Table S4). The colistin-resistant isolates were analyzed by API20E.

### 4.3. Antimicrobial Susceptibility Testing

For colistin-resistant isolates, the minimal inhibitory concentration (MIC) for colistin using the broth microdilution procedure according to the EUCAST breakpoints was determined [26]. The ESBL-producer isolates were determined by double disc synergy according to CLSI [27]. Additionally, the MIC of ampicillin (AMP), ceftazidime (CAZ), cefotaxime (CTX), imipenem (IMP), amikacin (AK), gentamicin (GEN), ciprofloxacin (CIP), nalidixic acid (NAL) and tetracycline (TET) were determined according to CLSI standards [27].

### 4.4. PFGE Analysis

The genetic relatedness of isolates was examined by pulsed-field gel electrophoresis (PFGE) [28]. The results were analyzed using GelCompar II software (Applied Maths, Kortrijk, Belgium).

### 4.5. Plasmid Profile Determination, Mating and Transformation Experiments

Plasmid DNA profiles were obtained from the isolates according to the method described by Kieser [29] *E. coli* NCTC 50192 plasmids 154-, 66-, 48- and 7-kb were used as molecular size markers [30]. The linear regression equation was used for molecular weight plasmid calculation. Colistin resistance transfer was evaluated by mating and transformation experiments [31,32]. The conjugation assays were performed using the *E. coli* strain J53-2 and co-selected with 100 μg/mL rifampicin and 32 μg/mL of potassium tellurite. Since the *ter* operon was identified in the genetic context of *mcr-1* (Supplementary Figure S1) and considering that colistin has a low diffusion in solid media, we decided to use tellurite resistance as a phenotypic selection marker in conjugation assays. On the other hand, the pRC2-007 plasmid was extracted using ion-exchange columns (Qiagen, Hilden, Germany). The plasmid was electroporated to *E. coli* DH10B (Amersham, Darmstadt, Germany) and *Klebsiella variicola* F2R9 [33]. Transformants were selected on agar plates supplemented with 8 µg/mL of colistin.

### 4.6. Whole Genome Sequencing and In Silico Analysis

Total genomic DNA was extracted and purified using the DNeasy Kit (Qiagen, Hilden, Germany). Whole-genome sequencing of bacterial and phage samples was generated using the Illumina (MiSeq) platform. Quality-based trimming was performed with the SolexaQA software and *de novo* assembly was done with SPAdes v3.1.1. The contigs were subjected to a scaffolding process with SSpace v2.0. Gene prediction and annotation were carried out using the bioinformatic MicroScope platform [34].

The Multilocus Sequence Typing (MLST), serotype and incompatibility group (Inc) of plasmids were determined in silico (http://www.genomicepidemiology.org, accessed on 15 October 2021) and prediction of closely related plasmids were obtained by BacWGSTdb (http://bacdb.cn/BacWGSTdb/, accessed on 5 July 2021) Virulence genes were determined by three platforms: VirulenceFinder 2.0 [35], VFDB [36] and BacWGSTdb [37] (http://bacdb.cn/BacWGSTdb/, accessed on 7 July 2021). Antimicrobial resistance genes were determined by ResFinder (http://www.genomicepidemiology.org, accessed on 15 July 2021) and BacWGSTdb (http://bacdb.cn/BacWGSTdb/, accessed on 12 October 2021). Phylogroup assignation was performed by the ClermonTyper web platform (http://clermontyping.iame-research.center, accessed on 17 October 2021) [38]. MobileElementFinder (http://www.genomicepidemiology.org, accessed on 25 October 2021) was used to detect mobile genetic elements associated with antibiotic resistance and virulence genes. The toxin-antitoxin systems HigAB and pdh/doc were screened by BLASTn search (https://blast.ncbi.nlm.nih.gov/Blast.cgi, accessed on 25 October 2021). Finally, the prophage structure was determined by the PHASTER program (https://phaster.ca, accessed on 25 October 2021). Plasmids and prophage with similar structures to the pRC2-007 were searched by BLASTn using >90% of nucleotide identity. Alignment of plasmids that contained the *mcr-1* gene inside a prophage and genetic structures of *mcr-1* was generated by Easyfig win_2.2 [39].

### 4.7. Comparative Analysis of mcr-1 E. coli Isolates

To explore the genetic relatedness between Mexican *mcr-1* positive isolates and isolates from other sources, we performed a core genome MLST (cgMLST) analysis. A minimum spanning tree (MST) was constructed including isolates with the same sequence type, closely related and positive to the *mcr-1* positive isolates which were identified by the BacWGSTdb 2.0 [37]. The threshold for cluster determination was 25 alleles according to BacWGSTdb 2.0; however, this cut-off point was not stringent when we could not find close relatives.

### 4.8. In Silico Identification of Phages and Prophages Carrying mcr-1 Genes

The *E. coli* and *K. pneumoniae* genomes were obtained from the RefSeq Database (https://www.ncbi.nlm.nih.gov/refseq/) accessed on 1 November 2020. The *mcr-1* gene was screened by the ggsearch36 program in all *E. coli* and *K. pneumoniae* genomes obtained from RefSeq Database (https://www.ncbi.nlm.nih.gov/refseq/) accessed on 5 November 2019. Subsequently, the prediction of phages and prophages was made in those genomes positive for *mcr-1* using the VirSorter program and the Refseqdb and Viromedb databases [40]. Finally, those genomes having the *mcr-1* gene within the phage or prophage prediction were selected and classified according to six categories (Cat). Cat-1 and Cat-2 correspond to intact and incomplete phages, respectively. Cat-3 and Cat-6 correspond to questionable phages or prophages and Cat-5 corresponds to incomplete prophages.

### 4.9. Bacteriophage Induction

The bacteriophage induction assay was carried out with mitomycin C. In 50 mL of fresh culture (at a cell density corresponding to 2 on the McFarland scale) of strain RC2-007, mitomycin C was added to a concentration of 0.5 μg/mL and the culture was incubated under 37 °C with shaking. The decrease in the absorbance at 600 nm was determined every hour for 6 h. Next, the supernatant was obtained by centrifugation at 3000× *g* for 12 min at 4 °C, was neutralized to pH 7.0 with 0.1N NaOH, and then filtered through a 0.22 μm pore membrane filter. Finally, supernatant containing bacteriophages was stored at 4 °C.

The assay was performed in triplicate. The phage DNA was extracted and purified using the DNeasy Kit (Qiagen, Hilden, Germany) and the *mcr-1* and a bacterial chromosomal control gene (*yjaA*) were screened by PCR using generic primers (Supplementary Table S4).

In addition, electron microscopy was carried out in a drop of medium containing the phage placed on a 200 mesh copper grid f coated with formvar/carbon (Electron Microscopy Sciences, Washington, PA, USA). The excess liquid was removed and the grid was negatively stained for 5 min with 2.5% uranyl acetate (aqueous solution) and washed several times with ultrapure water. Micrographs were obtained on a transmission electron microscope (JEOL 1011) operated at 80 kV.

### 4.10. Nucleotide Accession Numbers

The annotated genome sequences are available at the GenBank under the following accession numbers: JAIQXZ000000000 for *E. coli* C072, JAIQYA000000000 for *E. coli* C2033, JAIQYB000000000 for *E. coli* C2-107 and JAIQYC000000000 for *E. coli* C2-108.

## 5. Conclusions

In Mexico, there are few studies both in the clinical environment and in the food production chain that monitor resistance to colistin. This study reports the molecular and genomic traits of *mcr-1*-harboring *E. coli* from piglets. Our results show that colistin resistance followed independent acquisition pathways, but a common conjugative 170-kb plasmid was identified in the majority of isolates. The genetic relatedness of piglet *E. coli* isolates determined by cgMLST with non *mcr-1*-producing isolates derived from avian hosts supports the horizontal transfer of colistin resistance. The phage-like plasmid carrying *mcr-1* gene was less common in the farm. The structure of this phage-like plasmid was identified in other plasmids obtained from *E. coli* and *K. pneumoniae* recovered from non-clinical samples in China. The phages and prophages carrying *mcr-1* in *E. coli* and *K. pneumoniae* genomes could be closely related to the dissemination of the colistin resistance. The presence of CTX-M-type genes carried by the *mcr-1*-producing *E. coli* isolates implies the potential co-selection of *mcr-1* positive isolates with the use of cephalosporins. The co-occurrence of ESBLs and virulence genes in *mcr-1*-carrying isolates may favor reservoirs of clinically relevant antibiotic-resistant bacteria in the food production chain that influence human health.

## Figures and Tables

**Table 1:** Molecular characteristics of colistin-resistant *E. coli* isolates recovered from piglet fecal samples.

Bacterial Species	Isolate/ Transcojugant	Isolation Date Month/Year	Age of the Swine	Sex	RP	ESBL-Producer	*mcr-*Type Gene	Plasmid Profile (Kb) ^d^	Conjugation Frequency	CTX-M-Type	PFGE	MIC (µg/mL)									
												COL	AMP	CAZ	CTX	IMP	AK	GEN	CIP	NAL	TET
*E. coli*	C072	03/2015	12 days	Male	+	+	*mcr-1*	150, 170	NA	+	NR	4	>256	8	>32	1	2	16	1	>256	>64
	T-C072 ^a^	NA	NA		+	+	*mcr-1*	170	8.3 × 10^−8^	+	NA	2	256	4	>32	0.5	0.5	4	0.015	64	32
*E. coli*	C2033	09/2015	1 month	Male	+	+	*mcr-1*	75, 90, 170	NA	+	NR	4	>256	4	>32	1	2	32	2	>256	>64
	T-C2033 ^a^	NA	NA		+	+	*mcr-1*	170	1.2 × 10^−3^	+	NA	1	>256	4	>32	0.5	0.5	4	0.015	64	4
*E. coli*	C2-107	09/2015	6 days	Female	+	+	*mcr-1*	170	NA	+	A	4	>256	4	>32	0.5	2	16	4	>256	0.5
	T-C2-107 ^a^	NA	NA		+	+	*mcr-1*	170	3 × 10^−5^	+	NA	1	>256	16	>32	1	2	16	0.06	64	0.5
*E. coli*	C2-108	09/2015	6 days	Male	+	+	*mcr-1*	170	NA	+	A	4	>256	4	>32	0.5	2	16	4	>256	0.5
	T-C2-108 ^a^	NA	NA		+	+	*mcr-1*	170	2.1 × 10^−5^	+	NA	1	>256	16	>32	1	2	16	0.06	64	0.5
*E. coli*	RC2-007 ^b^	09/2015	2 months	Male	+	+	*mcr-1*	100, 120	NA	+	NR	4	>256	>32	>32	0.06	0.25	>32	16	>256	64
	TpEcoDH10B ^c^	NA	NA		+	-	*mcr-1*	100	NA	-	NA	2	ND	ND	ND	ND	ND	ND	ND	32	ND
*K. variicola*	F2R9	NA	NA		-	-	*-*	**-**	NA	-	NA	0.5	ND	ND	ND	ND	ND	ND	ND	ND	ND
	TpKvF2R9 ^c^	NA	NA		+	-	*mcr-1*	100	NA	-	NA	16	ND	ND	ND	ND	ND	ND	ND	32	ND

^a^*E. coli* J53-2 used in the mating experiments; which have rifampicin resistance and methionine and proline auxotrophies. ^b^ The colistin-resistant *E. coli* RC2-007 isolate that harbor the *mcr-1* gene in a phage-like plasmid (16). ^c^ Transformants of *E. coli* DH10B (TpEcoDH10B) and *K. variicola* (TpKvF2R9) acquiring the pRC2-007 plasmid from RC2-007 strain (16). ^d^ The underlined and boldface plasmids contain the *mcr-1* gene identified by mating and transformation experiments, respectively. Abbreviations: RP, Rapid Polymyxin test; NA, not applied; NR, nor related; ND, not determined; MIC, minimal inhibitory concentration.

**Table 2:** Typing results of *E. coli* isolates collected from piglets.

Isolate	Sequence Type a	Serotype a	Phylogroup b	Replicon Carrying the *mcr-1* Gene	Incompatibility Group (Inc) a	Genetic Context of *mcr-1* Gene c
C072	162	089:H19	B1	Plasmid	HI2, N, FIB	ISApl1-*mcr-1*-PAP2
C2-033	354	01:H34	F	Plasmid	HI2, N, FIB	ISApl1-*mcr-1*-PAP2
C2-107	1286	O16:H32	A	Plasmid	HI2, HI2A, N	ISApl1-*mcr-1*-PAP2
C2-108	1286	O16:H32	A	Plasmid	HI2, HI2A, N	ISApl1-*mcr-1*-PAP2
RC2-007	744	O89:H9	A	Plasmid/Prophage	Incp0111, FIB	ISApl1-*mcr-1*

^a^ The Multilocus Sequence Typing (MLST), resistome, serotype and incompatibility group (Inc.) of plasmids were determined in silico (http://www.genomicepidemiology.org, accessed on 1 June 2021). ^b^ Phylogroup was determined by the ClermonTyper web platform (http://clermontyping.iame-research.center, accessed on 5 June 2021). ^c^ Genetic context confirmed by PCR and sequencing. Plasmids or phage-like plasmid that carries the *mcr-1* gene are described in bold characters.

## Data Availability

Publicly available datasets were analyzed in this study. This data can be found here: https://www.ncbi.nlm.nih.gov/bioproject/PRJNA762933 and https://www.ncbi.nlm.nih.gov/biosample/SAMEA45274168 (accessed on 18 December 2021).

## Supplementary Materials

The following are available online at https://www.mdpi.com/article/10.3390/antibiotics11020157/s1, Table S1. Genomic features of *E. coli* isolates; Table S2. Resistome of colistin-resistant *E. coli* isolates; Table S3. Phages and prophages carrying *mcr-1* identified on *E. coli* and *K. pneumoniae* genomes; Table S4. Primers used for *mcr-*type gene identification; Figure S1. Genome map of prophage and the genetic context of *mcr-1* gene. The predicted genes and the functions predicted by PHASTER program [41] are indicated as boxes with the superscript number in each box. Figure S2. Electronic microscopy of phage that contained no detectable *mcr-1* gene; Figure S3. Comparison of phage-like plasmid that contains the *mcr-1* gene in the pRC2-007 plasmid with other phage-like plasmids. References [42,43,44] are cited in the supplementary materials.
