# Supplementary material for: Molecular and Genomic Insights of mcr-1-Producing Escherichia coli Isolates from Piglets
Source: Antibiotics (Basel). 2022 Jan 26;11(2):157. doi: 10.3390/antibiotics11020157 (PMC8868104; doi:10.3390/antibiotics11020157)
Supplement: Supplementary file 1 [file antibiotics-11-00157-s001.zip › antibiotics-1537940-supplementary.pdf]

Supplementary Table S1. Genomic features of *E. coli* isolates.

Genome	Contigs	Genome Length (bp)	G-C content (%)	Coding Sequence (CDS)	tRNA
**C072**	115	5168708	50.37	4913	59
**C2033**	107	5243891	50.31	4920	63
**C2-107**	147	5086635	50.50	4954	63
**C2-108**	139	5042928	50.52	4890	57

Supplementary Table S2. Resistome of colistin-resistant *E. coli* isolates.

Antibiotics family	Isolates and antibiotic resistance genes[a]				
	**C072**	**C2033**	**C2-107**	**C2-108**	**RC2-007**
**Aminoglycosides**	*aac(3)-IV, aadA2, aadA5, aph(4)-Ia, aph(6)-Id aph(3'')-Ib, aph(3')-Ia*	*aac(3)-IV, aaC2-II-d, aadA2, aph(4)-Ia, aph(3')-Ia*	*aph(4)-Ia, aac(3)-IV, aph(3')-Ia, aadA2, aadA1*	*aph(4)-Ia, aac(3)-IV, aph(3')-Ia, aadA2, aadA1*	*aac(3)-IIa, aadA5, aph(3'')-Ib, aph(6)-Id*
**Tetracyclines**	*tetB*	*tetB*	-	-	*tetB*
**Florfenicol/ chloramphenicol**	*cat, cmlA1, floR*	*cmlA1, floR*	*cmlA1, floR*	*cmlA1, floR*	*cat, floR*
**Trimethoprim**	*dfrA17*	*dfrA17*	-	-	*dfrA17*
**Fosfomycin**	*fosA3*	*fosA3*	*fosA3*	*fosA3*	-
**Macrolides**	*mphA*	*mphA*	*mphA, mdf(A)*	*mphA, mdf(A)*	*mphA*
**Sulfonamides**	*sul3*	*sul2, sul3*	*sul2, sul3*	*sul2, sul3*	*sul1, sul2, sul3*
**Lincosamide**	-	-	-	-	*inuF*
**-lactam**	CTX-M-14, TEM-1	CTX-M-14	CTX-M-14	CTX-M-14, TEM-116	CTX-M-55
**Colistin**	*mcr-1.1*	*mcr-1.1*	*mcr-1.1*	*mcr-1.1*	*mcr-1.1*
**Quinones/ Fluoroquinolones**	Ser83Leu and Asp87Asn for GyrA, and Ser80Iso for ParC	Ser83Leu and Asp87Asn for GyrA, and Ser80Iso for ParC	Ser83Leu and Asp87Asn for GyrA, and Ser80Iso for ParC	Ser83Leu and Asp87Asn for GyrA, and Ser80Iso for ParC	Ser83Leu and Asp87Asn for GyrA, and Thr56Ala and Ser80Iso for ParC

[a]Both chromosomal mutations of gyrases and topoisomerase as acquired resistance genes (coverage greater than 90% identified) were determined in the Center for Genomic Epidemiology using the ResFinder tool (http://www.genomicepidemiology.org).

Supplementary Table S3. Phages and prophages carrying *mcr-1* identified on *E. coli* and *K. pneumoniae* genomes.

**Bacterial species**	**Level**	**No. de genomes (%)**[a]	***mcr-1* positive (%)**	**VirSorter**[b]						**Genomes with prediction of intact and incomplete phages or prophages**
				Cat-1	Cat-2	Cat-3	Cat-4	Cat-5	Cat-6	
				Phage			Prophage			
E. coli										
	Complete	689 (5)	48	0	0	28	0	2	12	**Cat-5** (EC590 and HS30-1)
	Draft	12,868 (95)	820	10	12	313	0	2	202	**Cat-1** (2140, 2142, 3421, A351, B146, HU4, RC2-007, XJ4, YH17180 and YH17137), **Cat-2** (A200, A215, A245, A246, A276, A26, A38, A415, A6, HEB3, HLJ8 and LN48) and **Cat-5** (3768 and A432)
**Total**		13,557	868 (6.4)	10	12	341	0	4	214	
K. pneumoniae										
	Complete	306 (5)	3 (1)	0	0	2	0	0	1	no strains
	Draft	6,297 (95)	42 (0.7)	1	1	23	0	0	2	Cat-1 (SCKP-LL83) and Cat-2 (SCKP020138)
**Total**		6,603	45 (0.7)	1	1	25	0	0	3	

[a]Genomes obtained from RefSeq Database at November, 2019.
[b]Phages and prophages identified in the *E. coli* and *K. pneumoniae* using VirSorter program [40].

Supplementary Table S4. Primers used for *mcr-* and CTX-M-type genes identification.

Gene target	Primer and Sequence (5′ – 3′)	Amplicon (bp)	Reference
mcr-1	CLR5-F: GGTCAGTCCGTTTGTTC<br>CLR5-R: CTTGGTCGGTCTGTAGGG	309	[8]
mcr-2	MCR-12-F: TGGTACAGCCCCTTTATT<br>MCR-12-R: GCTTGAGATTGGGTTATGA	600 pb	This study
mcr-3	mcr3-F: TTGGCACTGTATTTTGCATTT<br>mcr3-R: TTAACGAAATTGGCTGGAACA	542 pb	[42]
mcr-4	Mcr-4 FW: ATTGGGATAGTCGCCTTTTT<br>Mcr-4RV: TTACAGCCAGAATCATTATCA	487 pb	[43]
mcr-5	MCR5_fw: ATGCGGTTGTCTGCATTTATC<br>MCR5_rew: TCATTGTGGTTGTCCTTTTCTG	1644 pb	[44]
**CTX-M**	CTX-MF: GCTGTTGTTAGGAAGTGTG<br>CTX-MR: GGTGACGATTTTAGCCGCC	811 bp	[30]

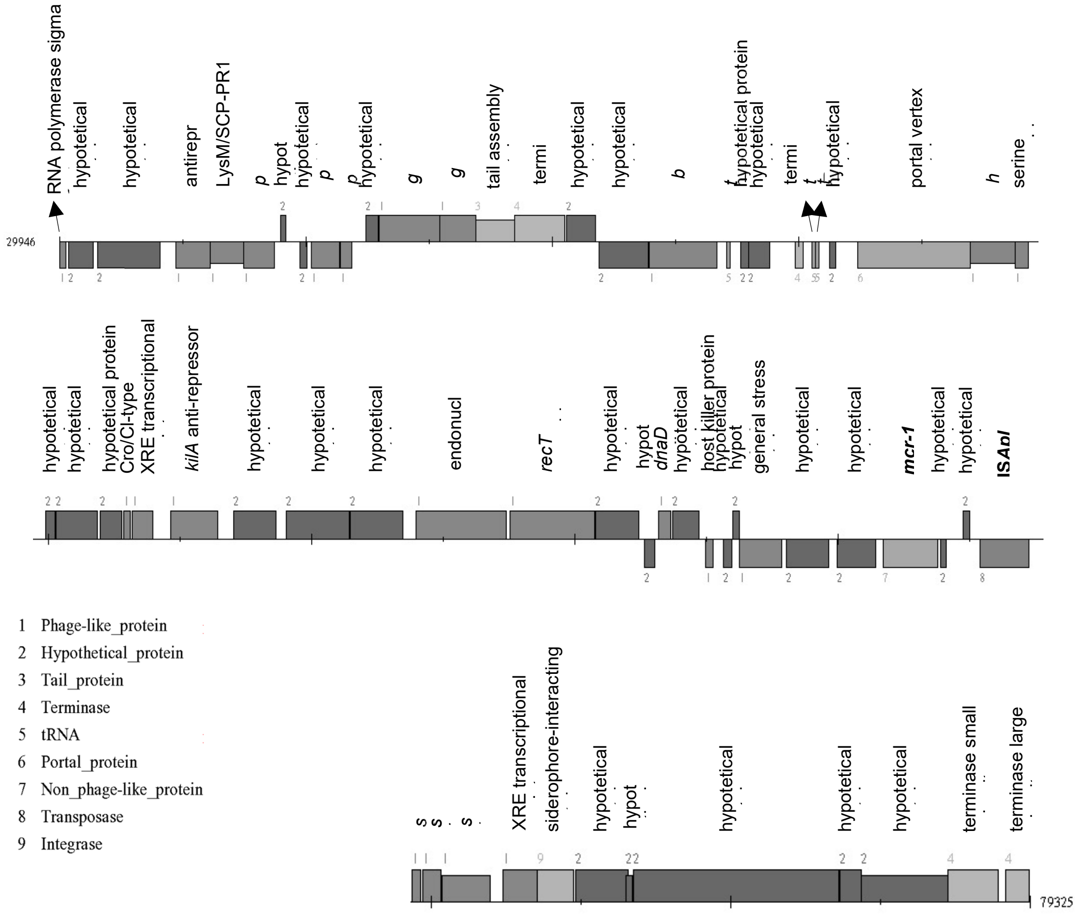

Supplementary Figure S1. Genome map of prophage as the genetic context of *mcr-1* gene. The predicted genes and the functions predicted by PHASTER program [41] are indicated as boxes with the superscript number in each box.

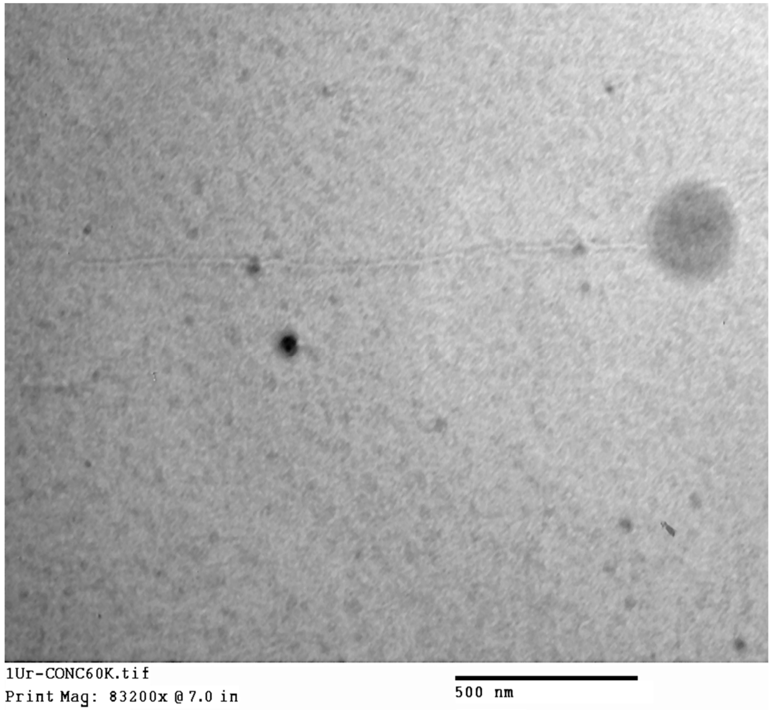

Supplementary Figure S2. Electronic microscopy of phage that contained no detectable *mcr-1* gene.

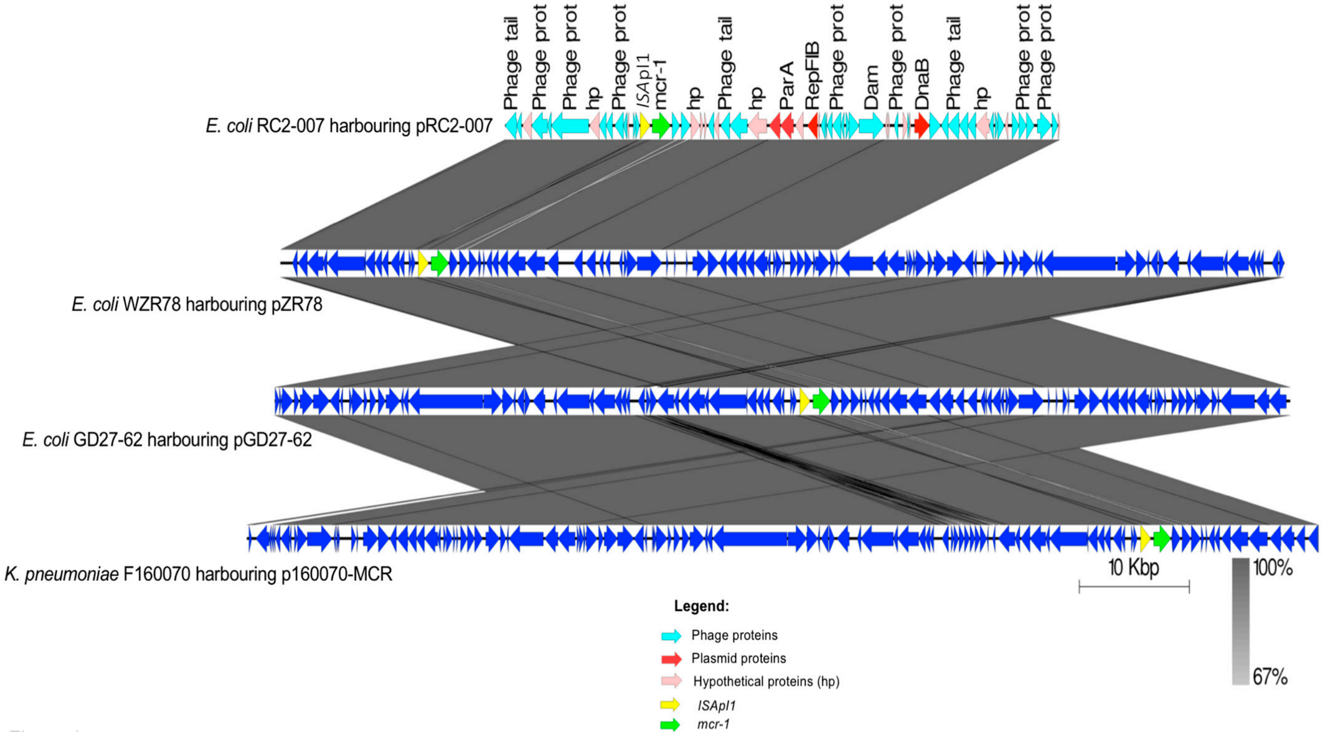

Supplementary Figure S3. Comparison of phage-like plasmid that contains the *mcr-1* gene in the pRC2-007 plasmid with other phage-like plasmids
